# *Burkholderia terrae* BS001 migrates proficiently with diverse fungal hosts through soil and provides protection from antifungal agents

**DOI:** 10.3389/fmicb.2014.00598

**Published:** 2014-11-11

**Authors:** Rashid Nazir, Diana I. Tazetdinova, Jan Dirk van Elsas

**Affiliations:** Department of Microbial Ecology, Centre for Ecological and Evolutionary Studies, University of GroningenGroningen, The Netherlands

**Keywords:** *Burkholderia terrae* BS001, mycelial protection, antifungal agents, cycloheximide, bacterial-fungal interactions

## Abstract

Soil bacteria can benefit from co-occurring soil fungi in respect of the acquisition of carbonaceous nutrients released by fungal hyphae and the access to novel territories in soil. Here, we investigated the capacity of the mycosphere-isolated bacterium *Burkholderia terrae* BS001 to comigrate through soil along with hyphae of the soil fungi *Trichoderma asperellum*, *Rhizoctonia solani, Fusarium oxysporum, F. oxysporum pv lini, Coniochaeta ligniaria, Phanerochaete velutina, and Phallus impudicus*. We used *Lyophyllum sp.* strain Karsten as the reference migration-inciting fungus. Bacterial migration through presterilized soil on the extending fungal hyphae was detected with six of the seven test fungi, with only* Phallus impudicus* not showing any bacterial transport. Much like with *Lyophyllum* sp. strain Karsten, intermediate (10^6^–10^8^ CFU g^-1^ dry soil) to high (>10^8^ CFU g^-1^ dry soil) strain BS001 cell population sizes were found at the hyphal migration fronts of four fungi, i.e., *T. asperellum*, *Rhizoctonia solani, F. oxysporum* and* F. oxysporum pv lini,* whereas for two fungi, *Coniochaeta ligniaria* and *Phanerochaete velutina*, the migration responses were retarded and population sizes were lower (10^3^–10^6^ CFU g^-1^ dry soil). Consistent with previous data obtained with the reference fungus, migration with the migration-inciting fungi occurred only in the direction of the hyphal growth front. Remarkably, *Burkholderia terrae* BS001 provided protection from several antifungal agents to the canonical host *Lyophyllum sp.* strain Karsten. Specifically, this host was protected from *Pseudomonas fluorescens* strain CHA0 metabolites, as well as from the anti-fungal agent cycloheximide. Similar protection by strain BS001was observed for *T. asperellum,* and, to a lower extent,* F. oxysporum* and *Rhizoctonia solani*. The protective effect may be related to the consistent occurrence of biofilm-like cell layers or agglomerates at the surfaces of the protected fungi. The current study represents the first report of protection of soil fungi against antagonistic agents present in the soil provided by fungal-associated *Burkholderia terrae* cells.

## INTRODUCTION

In many natural environments, bacteria and fungi live together in the same microhabitat (Johansson et al., 2004). In such cohabitations, the two partners may have developed strategies to interact with each other, to the mutual success of the interaction (Partida-Martinez and Hertweck, 2005; Partida-Martinez et al., 2007a,b). With basis in their mycelial way of growth, soil fungi are able to cross the air-filled gaps in natural soil (De Boer et al., 2005). Several soil fungi can also facilitate the movement of particular bacteria along the hyphal networks in soil, thus serving as fungal highways (Kohlmeier et al., 2005; Warmink and van Elsas, 2009; Warmink et al., 2011), and further translocate chemical substrates to distant places (serving as fungal pipelines; Furuno et al., 2012a; Banitz et al., 2013; Schamfuß et al., 2013). Concomitantly with the growth of fungi through soil, hospitable niches for the local bacterial communities in the microhabitat at the fungal surface are created (Nazir et al., 2010). Specific bacteria have thus evolved that benefit from such hospitable niches (Haq et al., 2014; Pion et al., 2013). Moreover, fungi proliferating in soil may also serve as agents that enrich and transport particular bacterial species, e.g., pollutant-degrading soil bacteria (Furuno et al., 2010, 2012b; Wick et al., 2007, 2010).

In recent years, soil-colonizing fungi and associated bacteria have gained renewed attention as consortia that may increase the biodegradation potential of soil for recalcitrant pollutants (Banitz et al., 2011a,b, 2012; Harms et al., 2011). Among the various bacterial species present in fungal-affected soil habitats, particular ones, e.g., members of the genus *Burkholderia,* have been found to be very successful in colonizing the fungal surfaces (Warmink and van Elsas, 2009). Such types may constitute key protagonists of fungal-associated bacterial groups (Partida-Martinez et al., 2007a; Warmink and van Elsas, 2009; Warmink et al., 2011; Nazir et al., 2012; Suárez-Moreno et al., 2012). In previous work, (Warmink and van Elsas, 2008), several *Burkholderia* spp., including *Burkholderia terrae*, were found to be key inhabitants of the soil underneath the mushroom foot of *Laccaria proxima* (denoted the mycosphere). Subsequently, similar *Burkholderia* types were found to be enriched in soil (mycosphere) that is colonized by the saprotrophic fungus *Lyophyllum sp.* strain Karsten (Warmink and van Elsas, 2009). Moreover, one key strain, *Burkholderia terrae* BS001, was found to be able to assist other (non-migrating) bacteria, like *Dyella japonica* BS003, to comigrate with the fungal host (Warmink et al., 2011). Recently, we found that this migration capacity is not restricted to only one species but is spread across several related species of the genus *Burkholderia* (Nazir et al., 2012). Interestingly, the capability of each *Burkholderia* species to migrate along with growing fungal hyphae was different in different soils (Nazir et al., 2012).

In a key study on bacterial-fungal symbiosis, Partida-Martinez et al. (2007c) discovered that a remotely related *Burkholderia* species, *Burkholderia rhizoxinica,* lives inside the ascomycete *Rhizopus microsporus* (Partida-Martinez et al., 2007c). Interestingly, the phytotoxins secreted by this fungus are actually produced by the endomycotic *Burkholderia rhizoxinica* (Partida-Martinez and Hertweck, 2005; Partida-Martinez et al., 2007c). A tight interaction between *Burkholderia rhizoxinica* and *Rhizopus microsporus* was shown to exist, as the fungus could not sporulate in the absence of the bacterium (Partida-Martinez et al., 2007c) and the bacterium utilized its type three secretion system (T3SS) to interact with the fungus (Lackner et al., 2009).

In spite of the ecological relevance of the interaction of *Burkholderia* spp. with soil fungi, we still ignore to what extent this capacity to “ride the fungal highway” is applicable to other fungal hosts, and also what the potential benefit of association with a bacterium like *Burkholderia terrae* BS001 may be for the latter. Here, we thus extend the previous work on the migration of *Burkholderia terrae* BS001 along with hyphae of *Lyophyllum sp*. strain Karsten through soil to a suite of other fungi of ecological relevance, like in biocontrol, pathogenicity and rotting of wood, and also to systems in which fungal-adverse conditions reign. We hypothesized that (1) the capacity of *Burkholderia terrae* BS001 to comigrate with the hyphae of soil fungi is generalistic rather than specific, and (2) soil fungi can obtain benefits from the bacterial cells that associate with their hyphae in terms of enhanced protection from adverse conditions.

## MATERIALS AND METHODS

### GROWTH AND MAINTENANCE OF MICROORGANISMS

The fungal strains used in this study, i.e., the basidiomycetous *Lyophyllum sp.* strain Karsten (DSM2979), *Rhizoctonia solani* AG3, *Trichoderma asperellum* 302, *Coniochaeta ligniaria* ATCC44981, *Phallus impudicus* PI, *Phanerochaete velutina* PV, *Fusarium oxysporum* Fo47, and* F. oxysporum pv. lini* Foln3 were routinely grown on OFA plates, prepared with 30 g of oat flake (local shop) and 15 g of agar (Duchefa, Haarlem, Netherlands) in demineralized (milliQ) water to 1 l, and sterilized at 121°C for 21 min. Once every 4 weeks, the fungal cultures were transferred to fresh OFA plates for maintenance.

The bacterial strains used in this study, i.e., *Burkholderia terrae* BS001, *Pseudomonas tolaasii* BS295, *Chryseobacterium aurantiacum* BS126, *Chryseobacterium joostei* BS181 and *Pseudomonas fluorescens* CHA0, were maintained as frozen cultures in 20% glycerol (-80°C). Working stocks were maintained on R2A (Becton, Dickinson and Company, Sparks, MD, USA) agar plates, normally at room temperature, which were streaked onto new plates every week to maintain cell viability. After each third transfer, bacterial cultures were re-established from the original -80°C stock.

### SOIL MICROCOSM EXPERIMENTS

#### Preparation of bacterial inocula

To prepare cell suspensions for inoculation, bacterial strains were grown overnight in 5 ml of Luria-Bertani (LB) broth (pH 7.0; Sigma-Aldrich, Haarlem) at 23°C, with shaking. The cells were spun down for 5 min at 5,000 ×*g*, washed and resuspended in 1 ml of sterile saline (0.85% w/vol NaCl). This procedure was repeated twice. The final cell suspensions were then set to an OD660 of 0.05 (containing an estimated 10^7^ cells ml^-1^, as evidenced using dilution plating on R2A agar). In total, 50 μl of each relevant bacterial suspension was used directly for inoculation of soil or agar in the migration experiments.

#### Bacterial migration through soil with growing fungal hyphae and protection against antifungal agents

The experimental set-up (in microcosms) served two purposes, i.e., (1) analysis of the comigration behavior of *Burkholderia terrae* BS001 with the hyphae of different fungal counterparts, and (2) assessment of the putative protective effect against antifungal agents of the presence of strain BS001 on the fungal partner. For both types of analyses, the microcosms consisted of three-compartment Petri dishes (Greiner Bio one, Frickenhausen, Germany), as described elsewhere (Warmink and van Elsas, 2009). Briefly, two of the compartments were filled with pre-sterilized [autoclaved (twice)] Gieterveen (G) soil (pH 4.8). The soil moisture contents corresponded to 60% of the water holding capacity, the soil bulk density to about 1.3 g/cm^3^. A layer of approximately 8 mm depth was established. The third, non-soil, compartment was filled with OFA and served as a nutrient source for the fungus (Figure S2). The physical barriers between the nutritive and soil compartments prevented compounds from the OFA compartment to reach the soil compartments. The barriers were overcome, though, by fungal hyphae growing out from the OFA compartment into the soil compartments, and hence mycelial fronts progressively extending into the soil were achieved. Triplicate set-ups were used for each of the treatments described in the following. The systems were inoculated, in separate, with each of the aforementioned fungi, by placing agar plugs with fungal mycelium on top of the OFA surface. The systems were incubated at 28°C, thereby allowing the colonization of the OFA compartment plus the first 1 mm of the soil (prior to introduction of the bacterial cells). In the experiments, the soil water contents were carefully maintained.

Following the first incubation period, washed suspensions (50 μl total) of cells of the inoculant bacterium, i.e., *Burkholderia terrae* strain BS001, were placed evenly in one 3-mm-wide streak in the soil compartment directly adjacent to (touching) the front of the growing fungal hyphae. Control treatments consisted of the addition of BS001 cells in a similar streak to soil compartments of microcosms without fungal mycelium, and of sterile water added to the fungal-plus microcosms in a similar streak.

For the experiments on co-migration of strain BS001 with different fungal mycelia, the microcosm systems inoculated with both partners were incubated at 23°C under constant moisture level, and sampled after 9, 12, 15, and 18 days of inoculation.

For the experiments on the effect of strain BS001 on fungal outgrowth in the presence or absence of antifungal agents in the soil, selected antifungal agents, including cycloheximide (CH), were applied to the soil compartments at a position about 15 mm away from the mycelial growth front. Following this inoculation, the microcosm systems were incubated at 23°C, taking care that the soil moisture contents remained at the initial levels. Visual observation of the systems, recording fungal growth, was then performed at regular time intervals, normally every day.

#### Microscopic observations of the interaction of *Burkholderia terrae* BS001 with different soil fungi

To observe the interactions of *Burkholderia terrae* BS001 with the selected fungi, it was marked with a gene expressing the green fluorescent protein (GFP) using a standard procedure on the basis of plasmid pKH2 (Roelants et al., 2002). In several selected colonies, the gene was found to express very well, as clear green cells could be observed by fluorescence microscopy, whereas the wild-type strain (used as the control) remained uncolored. This *gfp-*tagged *Burkholderia terrae* BS001 strain was then used in the observational work on the hyphal-associated BS001 cells.

Confirming previous work, all tests for the presence of *Burkholderia terrae* BS001 on soil-extracted mycelium consistently yielded negative results, due to the difficulty of obtaining sufficient soil-exposed fungal biomass. To assess the bacterial association with fungal hyphae, we then placed pre-sterilized microscope slide cover slips on the G soil surfaces in the microcosms and let the fungus grow over it (using systems with or without the *gfp*-marked BS001 cells). At several time points following bacterial inoculation, the cover slips were aseptically removed from the systems and subsequently observed by microscopy. For this purpose, an epifluorescence microscope (Zeiss Axiostar Plus) was used, with light of the excitation wavelength entering the sample through the objective lens and the fluorescence emitted by the sample coming to the detector by the same objective used for the excitation.

### BACTERIAL POPULATION DYNAMICS IN THE SOIL MICROCOSMS

Using triplicate systems per treatment, bacterial CFU counts were performed at regular time intervals following bacterial inoculation (see above). For this, samples (about 100 mg) were punched out (using a 4-mm diameter auger) at different sites within the soil compartments. The sites were selected to cover the migration front, the inoculation site as well as other specified positions in the soil compartments. The soil samples were processed for further analyses by serial tenfold dilution followed by plating onto selected agar media, i.e., R2A (general bacterial count), Gould’s S1 (Gould et al., 1985; count of fluorescent pseudomonads, i.e., strain CHA0), and/or PCAT (*Burkholderia* count; Salles et al., 2005). Plating was followed by incubation of the plates at 28°C and CFU counting after incubation for 1–5 days. CFU counts were transformed to counts per g dry soil, indicating the development of population densities of both the *Burkholderia terrae* and the antifungal bacteria.

### TESTING OF GROWTH OF *Burkholderia terrae* BS001 ON CYCLOHEXIMIDE

To evaluate if *Burkholderia terrae* BS001 can grow on CH as the sole C source, we prepared triplicate 100-ml Erlenmeyer flasks with 20 ml minimal medium (MM) supplemented with CH (0–0.5%) as the carbon and energy source. As a positive control, MM supplemented with 0.5% glucose was used. Strain BS001 was then introduced as 10^6^ (pre-grown and washed) cells/ml into the media, after which flasks were incubated at 28°C (with shaking). Growth was measured by visual observation, OD determinations and CFU counting following dilution plating onto R2A plates.

### OFA PLATE INHIBITION EXPERIMENTS

The protective effect conferred by *Burkholderia terrae* BS001 upon *Lyophyllum sp.* strain Karsten and other selected fungi (see results) against the antagonistic pressure exerted by CH was tested. Different concentrations of CH, i.e., 6, 12.5, 25, 50, 75, and 100 μg/ml, were applied to fresh OFA plates. Moreover, ‘induced’ or ‘uninduced’ *Pseudomonas fluorescens* strain CHA0 metabolites (produced as detailed below) were also added. Unamended OFA plates served as controls. All plates were inoculated as follows. Cells of *Burkholderia terrae* BS001 (about 10^6^ per ml; 50 μl) and of selected fungi (plugs) were both placed, either in monoculture or in combination, at specified sites (see Figure S1A) on the plates. This yielded three combinations, i.e., ‘fungus-alone,’ ‘bacterium-alone,’ and ‘fungus + bacterium.’ The plates were incubated at 28°C and growth of the fungal mycelium over the plate was monitored over time, taking daily measurements, with reference time points at 0, 1, 7, 14, 21, and 28 days following introduction. On the basis of the collective data per treatment, the fungal migration ‘progress’ was calculated in relation to the presence or absence of the bacterial partner (expressed as mm per day). To monitor the bacterial population densities, the systems were sampled, at regular times, by removal of a small core from the OFA plates and vortex-mixing it for 15 min in 1 ml sterile saline. The resulting suspensions were then serially (10-fold) diluted and appropriate dilutions plated onto R2A to subsequently enumerate the CFU after 2–4 days of incubation at 23°C.

#### Production of a *Pseudomonas fluorescens* strain CHA0 metabolome

*Pseudomonas fluorescens* strain CHA0 metabolites were collected following overnight (approximately 15–16 h) growth of strain CHA0 (inoculum 10^4^–10^6^ cells) at 23°C in LB, either singly or in the presence of *T. asperellum* 302 (see Figure S1A). As a negative control, LB without any inoculation, incubated in the same conditions, was used. Cells were spun down from the grown culture to obtain supernatants. These supernatants, constituting the strain CHA0 metabolome, were then filter-sterilized (0.45 μm pore size) and used as supplements of the OFA medium, using 1:1 mixing with liquid OFA. In one experiment, the metabolites were thus homogeneously available in the whole plate, while in an additional experiment strain CHA0 metabolites (1:1 with medium) were introduced in a stripe, 15 mm away from the fungal inoculation site (see Figure S1B). The fungal growth patterns and bacterial dynamics, from both experimental set ups, were then monitored as explained above.

### STATISTICS AND DATA ANALYSIS

All experiments were performed using (at least) triplicate systems per treatment. Several experiments were repeated in time (see Results). At each time point, the (CFU) data were log-transformed, after which average values and standard deviations were calculated (shown between brackets in the text and/or as error bars in the figures).

Comparisons were made by statistical tests (*t*-test, ANOVA) using the SPSS package (SPSS, IBM, Statistics 1.8 for Windows), and data are reported as significant at *P* < 0.05.

## RESULTS

### GROWTH OF FUNGAL STRAINS THROUGH G SOIL MICROCOSMS IN RELATION TO THE PRESENCE OR ABSENCE OF *Burkholderia terrae* BS001

From the start of the experiments, all fungi, i.e., *Lyophyllum sp.* strain Karsten, *Rhizoctonia solani* AG3, *Trichoderma asperellum* 302, *Coniochaeta ligniaria* ATCC 44981, *Phallus impudicus PI, Phaenerochaete velutina PV, Fusarium oxysporum* Fo47 and *F. oxysporum pv. lini* Foln3 grew steadily as a hyphal front over the OFA compartment, and from that into the G soil compartments. Subsequently, the fungal hyphal fronts, visualized by mostly whitish mycelium at the soil surface, extended progressively through the soil compartments until reaching the edges of the plates. It should be noted that the movement of the two *Fusarium* spp. could be monitored for up to 20 mm, after which the growth fronts became very difficult to visually detect. Hyphal extension was irreverent of the presence of *Burkholderia terrae* BS001 cells, as for none of the fungi, any noticeable or significant inhibition of growth by the presence of *Burkholderia terrae* BS001 was found, as compared to the controls (*P* > 0.05). As judged from observing the rates of migration of the hyphal fronts, the growth patterns of the fungi through the soil compartments were significantly different for several of the species tested. Thus *T. asperellum* 302 exhibited the highest growth rate, i.e., 4.5 ± 0.2 mm/day, while* Phaenerochaete velutina* PV was the slowest grower, i.e., 0.8 ± 0.1 mm/day. The other fungi, i.e.,* Lyophyllum sp.* strain Karsten,* Coniochaeta ligniaria* ATCC44981, *Rhizoctonia solani* AG3 and both* Fusarium spp.* (Fo47 and Foln3), showed intermediate growth rates, i.e., 1.6 ± 0.24, 1.14 ± 0.16, 1.28 ± 0.13, and 1.5 ± 0.11 mm/day, respectively. Finally, *Phallus impudicus* PI grew only very poorly into the soil matrix, with a rate close to zero over experimental time.

### MIGRATION OF *Burkholderia terrae* BS001 ALONG WITH THE HYPHAL GROWTH FRONTS THROUGH G SOIL

As expected, *Burkholderia terrae* BS001 migrated along with the developing hyphae of *Lyophyllum sp.* strain Karsten (used as the reference fungus) through the G soil, establishing a population density of over 10^8^ CFU/g dry soil at the migration front (18 days; see **Table 1**). With six out of the seven other fungi, strain BS001 showed similar behaviour, i.e., migration with the developing fungal hyphae in the canonical growth direction of the fungi but not in the reverse direction (densities below the detection limit), albeit with different cell densities at the end of the experimental period at the migration fronts. For example, *F. oxysporum* Fo47 also supported >10^8^ CFU/g dry soil of *Burkholderia terrae* BS001 at the hyphal fronts, while *Rhizoctonia solani* AG3, *T. asperellum* 302 and *F. oxysporum* pv* lini* Foln3 supported population densities of 10^6^–10^8^ CFU/g dry soil. These densities apparently constituted the carrying capacities of the fungus/soil combinations in the microcosm systems used. In this respect, *Burkholderia terrae* BS001 showed migration to low densities during the same time course (18 day) at the migration fronts (i.e., in the range 10^3^–10^5^ CFU/g dry soil) of *Coniochaeta ligniaria* ATCC44981 and *Phaenerochaete velutina* PV. These numbers were significantly lower than those found with the aforementioned fungi (*P* < 0.05). Finally, *Burkholderia terrae* BS001 could not be shown to firm a clear association, resulting in comigration, with the (poorly migrating) *Phallus impudicus* PI hyphae.

**Table 1:** Migration of *Burkholderia terrae* BS001 through soil along the hyphae of different fungi.

Fungal species	Ecological feature	Hyphal type	Level of migration* (CFU/g dry soil)
*Lyophyllum sp*. strain Karsten	Saprotroph	Thread-like mycelia	+++
*Rhizoctonia solani* AG3	Phytopathogen	Thread-like mycelia	++
*Trichoderma asperellum 302*	Biocontrol	Thread-like mycelia	++
*Fusarium oxysporum* Fo47	Phytopathogen	Thread-like mycelia	+++
*F. oxysporum pv. lini* Foln3	Biocontrol	Thread-like mycelia	++
*Coniochaeta ligniaria ATCC 44981*	Saprotroph, avid organic matter degrader	Thread-like mycelia	+
*Phallus impudicus* PI	Wood rot	Cords	-
*Phaenerochaete velutina* PV	Wood rot	Cords	+

*+++, 10^8^ CFU *g*^-1^ dry soil at migration front; ++, 10^6^–10^8^ CFU *g*^-1^ dry soil at migration front; +, 10^3^–10^6^ CFU *g*^-1^ dry soil at migration front; -, <10^3^ CFU g^-1^ dry soil at migration front.*

### ASSOCIATION OF *Burkholderia terrae* BS001 CELLS WITH FUNGAL HYPHAE

**FIGURE 1:**
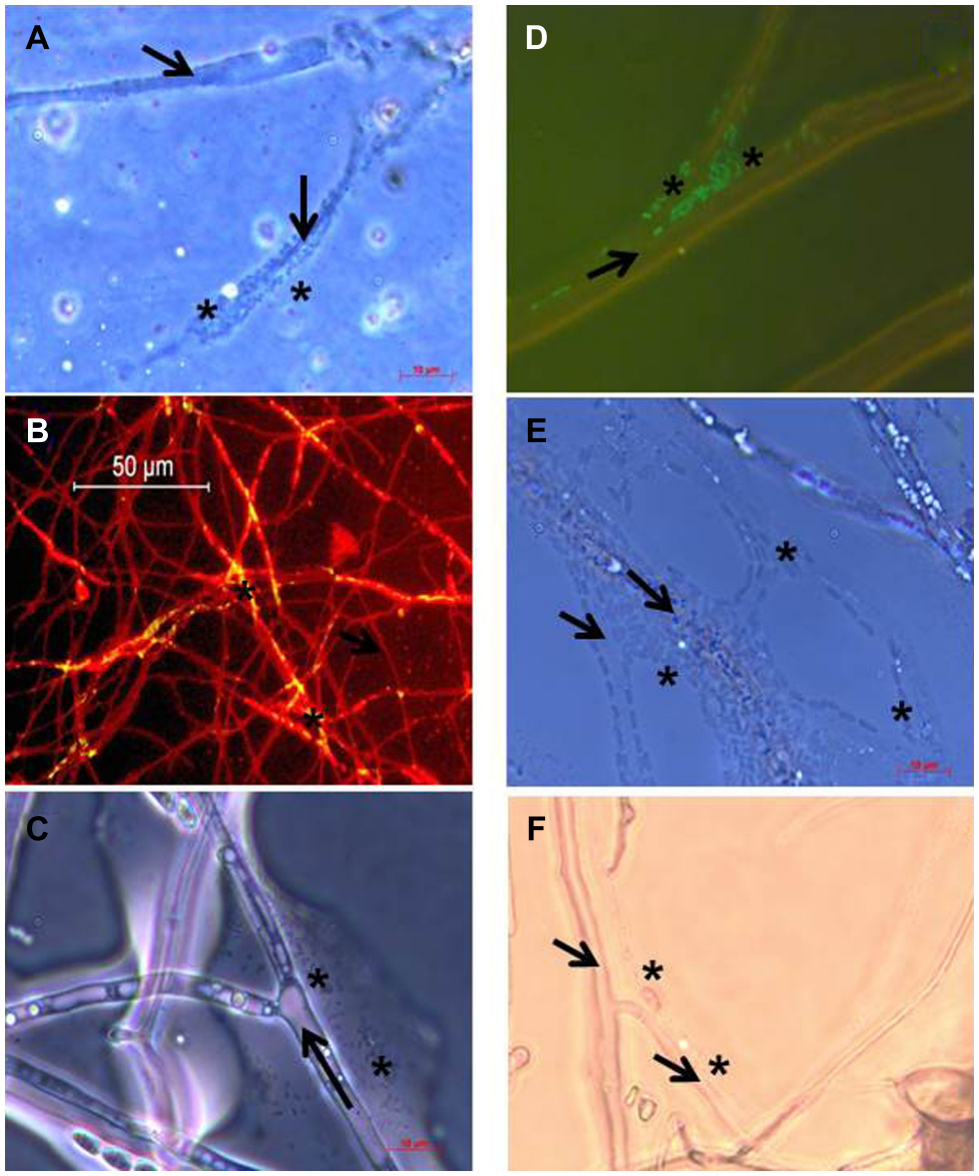
**Microscopic images of the association of *Burkholderia terrae* BS001 cells with mycelium of: **(A)***Phaenerochaete velutina* PV, **(B)*** Trichoderma asperellum 302,***(C)*** Rhizoctonia solani* AG3, **(D)***Fusarium oxysporum* Fo47, **(E)*** Coniochaeta ligniaria* ATCC44981 and **(F)*** Lyophyllum sp.* strain Karsten.** Arrows indicate the fungal hyphae, and asterisks indicate the presence of bacterial cell agglomerates. Microscopic observation was supported by differential staining with FITC (fungus) and acridine orange (strain BS001; **B**) or the green fluorescent protein (GFP; **C**).

As outlined in Materials and Methods, revealing the presence of *Burkholderia terrae* BS001 biofilms or cell agglomerates directly on soil-dwelling mycelium was technically too demanding. Using the cover slip soil incubation method instead (4–5 days following the start of the experiment), *gfp*-marked (or unmarked) *Burkholderia terrae* BS001 cells could be shown to become associated, as layers or agglomerates of cells, with the growing hyphae away from the inoculation sites of all fungi except* F. oxysporum* pv *lini* Foln3 and *Phallus impudicus* PI. These observations were consistent with the positive strain BS001 CFU counts. Thus, the mycelial networks in soil of *Phaenerochaete velutina* PV, *T. asperellum* 302,* Rhizoctonia solani* AG3, *F. oxysporum* Fo47, and *Coniochaeta ligniaria* ATCC44981, next to the reference fungus *Lyophyllum* sp. strain Karsten, had all been colonized by strain BS001 cells. The levels of colonization were diverse for the different fungal hosts, with either layers of cells (e.g., *T. asperellum* 302) or isolated cell agglomerates (e.g., *F. oxysporum* Fo47) being visible. **Figure 1** shows the colonization of the growing hyphae of the selected fungi by *Burkholderia terrae* BS001 cells.

### PROTECTIVE EFFECT OF *Burkholderia terrae* BS001 TO HOST FUNGI

#### Protection provided by *Burkholderia terrae* BS001 to *Lyophyllum* sp. strain Karsten against antifungal agents in soil

First, the selected strains *Pseudomonas fluorescens* CHA0, *Pseudomonas tolaasii* BS295, *Chryseobacterium aurantiacum* BS126, and *Chryseobacterium joostei* BS181 revealed to be antagonistic (growth-inhibiting), much like CH, to *Lyophyllum* sp. strain Karsten growing in dual-plate assays (data not shown). These four bacterial strains as well as the antifungal agent CH were thus used in a soil microcosm set-up.

In the microcosm compartments containing the presterilized G soil, the population densities of all antagonists, introduced at the onset about 15 mm away from the *Lyophyllum sp.* strain Karsten mycelial fronts, persisted at about the inoculum levels, i.e., about 10^6^ CFU per g dry soil, over the experimental time. Remarkably, *Lyophyllum sp.* strain Karsten was not able to grow over the sites where these bacterial antagonists had been applied (shown after 15 days; Figure S2), whereas it grew ‘normally’ through control G soil without the added antagonists. However, in all cases in which *Burkholderia terrae* BS001 had been introduced at the young mycelial fronts (about 5 × 10^6^ cells per g of soil), the fungal soil invader was able to grow over the soil zone where the antagonists were present (Figure S2), albeit in a retarded fashion (*P* < 0.05) as compared to the non-antagonist controls (see **Table 2**). Similar findings were obtained when the mycelial growth fronts were confronted with 25 μg/g dry soil of CH in the soil. Overall, the protective effect of *Burkholderia terrae* BS001 to *Lyophyllum sp.* strain Karsten was most prominent with *Chryseobacterium aurantiacum* BS126, as the fungal growth rate over the antagonistic zone was highest, i.e., 1.57 ± 0.10. Lower protective effects were seen with the other antagonists as well as with CH (**Table 2**). In the case of strain BS126, the fungal growth rates with strain BS001 and over the antagonistic regions were similar (*P* > 0.05) to those of the controls (no antagonist, with or without strain BS001). With respect to the other antagonists (including CH), the presence of *Burkholderia terrae* BS001 significantly lifted the fungal growth rates (*P* < 0.05), ‘rescuing’ the fungus. However, this resulted in a reduced fungal growth rate as compared to the controls (*P* < 0.05).

**Table 2:** Effect of *Burkholderia terrae* BS001 on the migration of *L. sp*. strain Karsten through soil as affected by the presence/absence of antifungal agents (bacterial strains and CH).

Antifungal agent present		Control	CH	BS126	BS181	BS295	CHA0
No	without BS001	1.66 (0.06)^a^	1.57 (0.07)^a^	1.55 (0.03)^a^	1.64 (0.07)^a^	1.68 (0.07)^a^	1.57 (0.03)^a^
	with BS001	1.60 (0.06)^a^	1.62 (0.07)^a^	1.57 (0.03)^a^	1.68 (0.07)^a^	1.64 (0.07)^a^	1.60 (0.06)^a^

Yes	without BS001	1.64 (0.03)^a^	ND^b^	ND^b^	ND^b^	ND^b^	ND^b^
	with BS001	1.68 (0.03)^a^	1.15 (0.10)^c^	1.57 (0.10)^a^	1.31 (0.10)^c^	1.31 (0.10)^c^	1.26 (0.17)^c^

*Average fungal growth rates (mm/d) with standard deviations (between brackets) are shown, measured over 15 days. Control, microcosm without any bacterial/antagonist application; CH, cycloheximide (25 μg/g dry soil); BS126, *Chryseobacterium aurantiacum* strain BS126*;* BS181, *Chryseobacterium joostei* strain BS181; BS295, *Pseudomonas tolaasii* strain BS295; CHA0, *Pseudomonas fluorescens* strain CHA0. ND, migration not detected. a, b, and c, statistical classes (different at *P*< 0.05).*

#### Protective effect of *Burkholderia terrae* strain BS001 against the *Pseudomonas fluorescens* strain CHA0 metabolome

**FIGURE 2:**
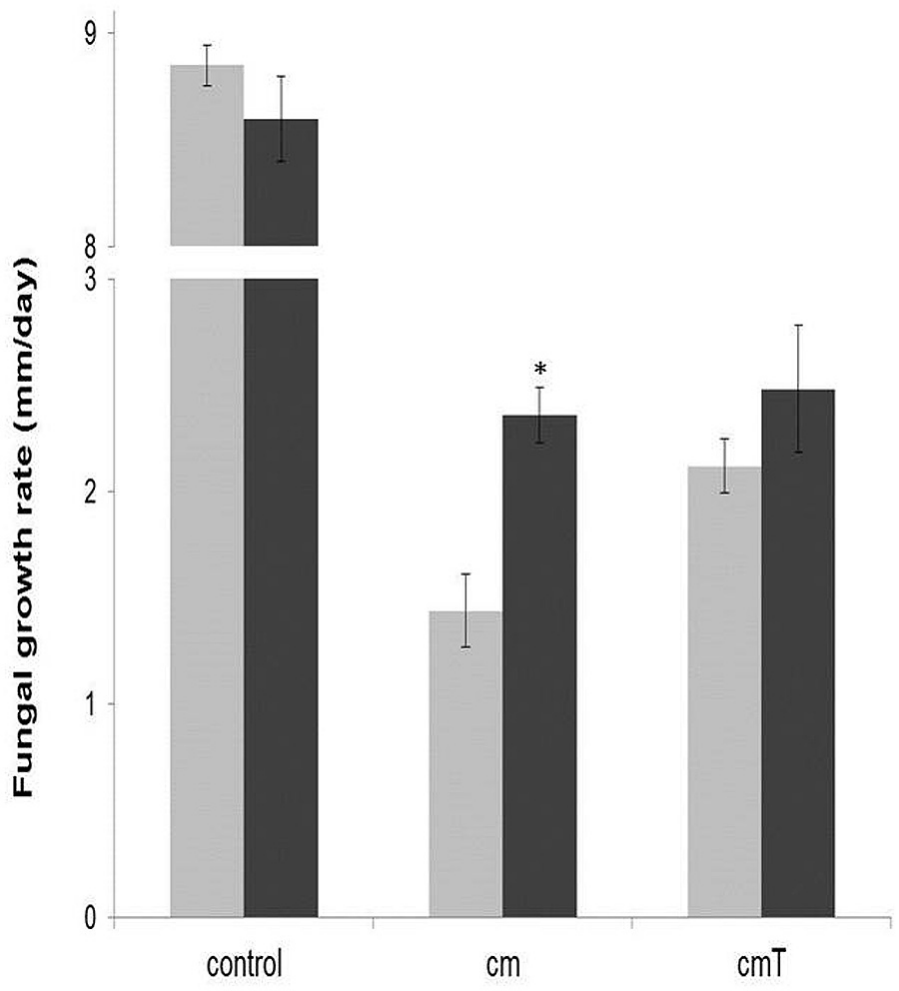
**Effect of the presence of *Burkholderia terrae* strain BS001 on the growth rate of *Lyophyllum sp.* strain Karsten on OFA supplemented or not with the *Pseudomonas fluorescens* CHA0 metabolome (measured over 5 days after the onset of the experiment).** control = unsupplemented OFA; cm = OFA supplemented with strain CHA0 metabolites; cmT = OFA supplemented with metabolites of strain CHA0 grown in the presence of *T. asperellum* 302. Gray bars: fungus alone; black bars: fungus growing in the presence of *Burkholderia terrae* BS001. Asterisk indicates a significant raise of fungal growth rate in the presence of strain BS001 (*P* < 0.05).

Strain CHA0 is a known antagonist of soil fungi, and it inhibited *Lyophyllum sp*. strain Karsten in a dual-plate assay. **Figure 2** shows the results of OFA plate experiments with the strain CHA0 metabolome. The presence of metabolites of both singly grown and ‘*T. asperellum* 302-grown’ *Pseudomonas fluorescens* CHA0 significantly inhibited the growth of *Lyophyllum sp.* strain Karsten on OFA, as compared to growth on unamended OFA (*P* < 0.05). Moreover, the presence of *Burkholderia terrae* BS001 cells on the OFA amended with singly grown strain CHA0 metabolites incited a significant (*P* < 0.05) increase in the growth rate of *Lyophyllum sp.* strain Karsten on this medium, as compared to its growth without BS001 (**Figure 2**, treatment cm). Whereas a trend toward a similar effect was found for metabolites from strain CHA0 grown in the presence of *T. asperellum* 302, this was not significant (*P* > 0.05).

**FIGURE 3:**
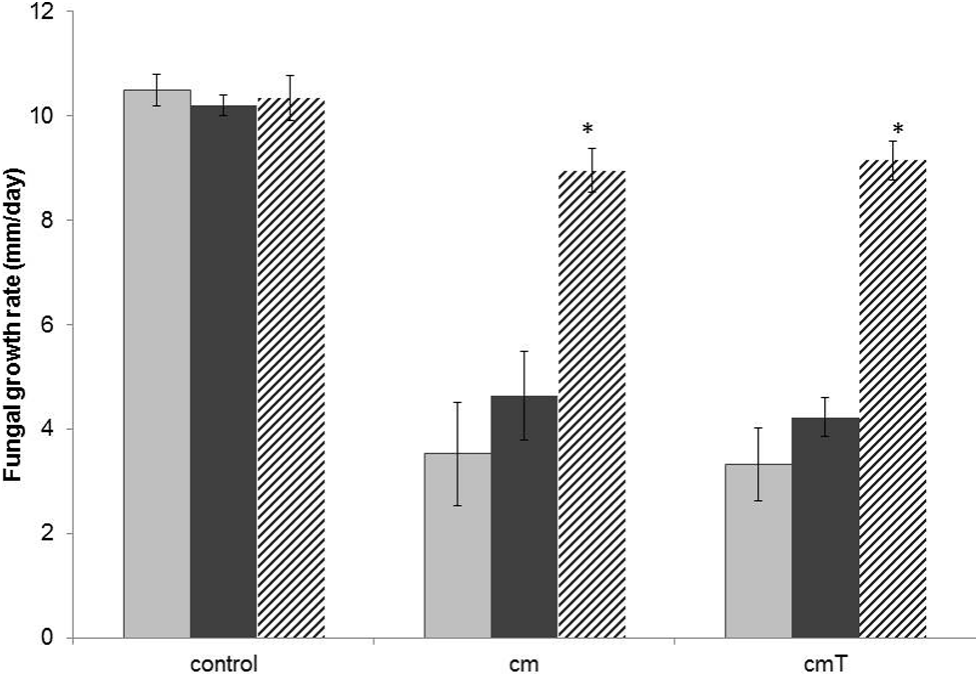
**Effect of the presence of *Burkholderia terrae* strain BS001 on the growth rate of *T. asperellum* 302 on OFA supplemented or not with the *Pseudomonas fluorescens* CHA0 metabolome (measured over 4 days after the onset of the experiment).** Explanation: control = unsupplemented OFA; cm = OFA supplemented with strain CHA0 metabolites (singly grown CHA0); cmT = OFA with metabolites of strain CHA0 grown in the presence of *T. asperellum* 302. Gray bars: fungus alone; black bars: fungus growing in the presence of *Burkholderia terrae* BS001. Striped bars: fungus growing in the presence of *Burkholderia terrae* BS001, with *Burkholderia terrae* BS001 introduced at the hyphal front 24 h after the onset of the experiment. Asterisks: significant raises of growth rates in the presence of strain BS001 (*P* < 0.05).

In the light of the probably tight interaction of *Burkholderia terrae* BS001 with the biocontrol fungus *T. asperellum* 302 (**Figure 1**), we extended the analysis of the potential protective effect exerted by *Burkholderia terrae* BS001 to this fungus. Indeed, observations made after both 4 and 5 days of incubation indicated that *T. asperellum* 302 was significantly inhibited in its outgrowth on OFA plates in the presence of strain CHA0 metabolites obtained from singly grown or *T. asperellum* 302 grown strain BS001 cells (*P* < 0.05; **Figure 3**). Remarkably, the protective effect of *Burkholderia terrae* BS001 to this fungal host was only significant when *Burkholderia terrae* BS001 cells were introduced at the fungal hyphae with a 1-day delay (*P* < 0.05), whereas no significant effect was detected in systems where *Burkholderia terrae* BS001 had been co-inoculated with the fungus (**Figure 3**). We further observed a reduction of spore formation (green zones within the *T. asperellum* 302 older mycelium) on OFA amended with the strain CHA0 metabolome as compared to the controls. This effect was stronger with metabolites from *T. asperellum* 302 – grown CHA0 cells than with those from the singly-grown CHA0 cells (Figure S3), indicating a possible sporulation-inhibiting effect of its metabolites. Interestingly, the presence of strain BS001 at the hyphal network alleviated the effect of such metabolites on sporulation of *T. asperellum* 302. Figure S3 contains the details of this observation.

#### Protective effect of *Burkholderia terrae* strain BS001 against cycloheximide

***Effect on *Lyophyllum* sp. strain Karsten and *T. asperellum* 302.*** First, we investigated whether *Burkholderia terrae* BS001 could grow in MM with CH as the sole C source, thus diminishing or abolishing the inhibitory action of CH. The BS001 cell densities (as CFUs) remained roughly at the initial level even after 1 month of incubation at 28°C (shaking). In the positive control flasks (MM supplemented with 0.5% glucose), strain BS001 showed vigorous growth after only 2 days at 28°C. Thus, *Burkholderia terrae* BS001 was not found to grow at the expense of CH in MM, which rules out the possibility of CH removal but leaves that of transformation into a (possibly less toxic) product.

**FIGURE 4:**
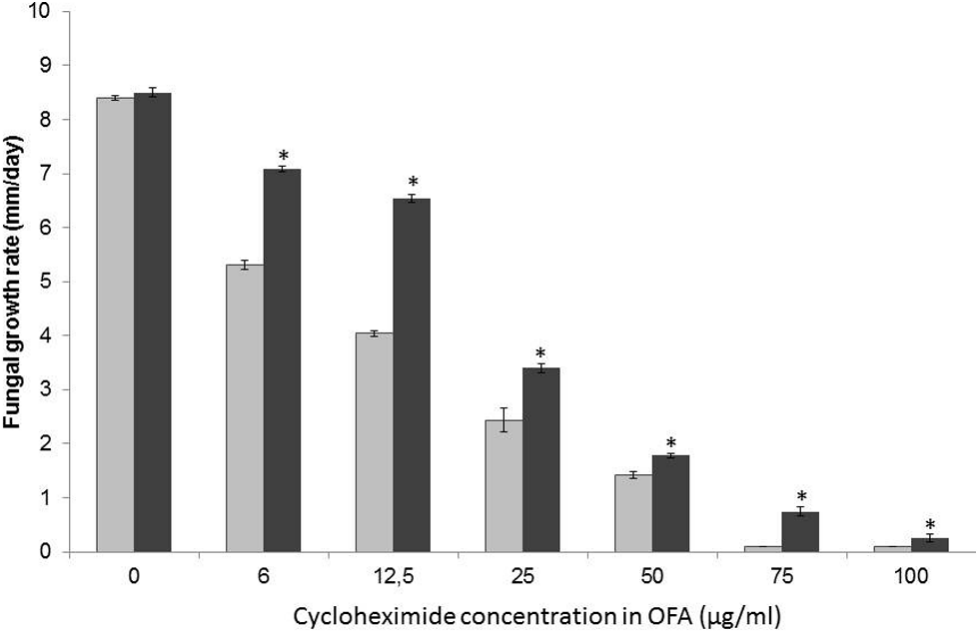
**Effect of the presence of *Burkholderia terrae* strain BS001 on the growth rate of *Lyophyllum sp.* strain Karsten on OFA supplemented or not with different concentrations of CH.** Data represent observations after 25 day of incubation; growth at 75 and 100 ug/ml CH was recorded as from day 21. Gray bars: fungus alone; black bars: fungus growing in the presence of *Burkholderia terrae* BS001. Asterisks: significant raises of fungal growth rates in the presence of strain BS001 (*P* < 0.05).

The potential of *Burkholderia terrae* BS001 to protect *Lyophyllum sp.* strain Karsten from CH was then tested using a CH concentration range (0–100 μg/ml) in OFA plates. Unamended OFA allowed fungal outgrowth, from the inoculation site, at about 8.5 mm/day both without and with strain BS001 cells (**Figure 4**), whereas the presence of CH in all tested concentrations was clearly detrimental to hyphal outgrowth. The inhibitory effects exerted by CH were significant in all cases and became progressively stronger with increasing CH levels (*P* <0.05). Specifically, 6 and 12.5 μg/ml CH reduced the growth rates to 5.31 ± 0.08 and 4.07 ± 0.03 mm/day, respectively. CH levels of 25 and 50 μg per ml further reduced these rates to 2.44 ± 0.22 and 1.42 ± 0.02 mm/day. The presence of 75 and 100 μg of CH per ml of OFA inhibited fungal growth completely, and growth arrest lasted for up to 3 months following inoculation (**Figure 4**).

Very interestingly, the presence of *Burkholderia terrae* BS001 cells (introduced at about 10^6^–10^7^ CFU) adjacent to the *Lyophyllum sp.* strain Karsten hyphal front significantly reduced the inhibitory effect of CH in the OFA on fungal growth. Although different in magnitude, the effect was significant (*P* < 0.05) for all used CH concentrations (**Figure 4**, indicated by asterisks). Specifically, the fungal growth rates were 5.31 ± 0.03, 4.047 ± 0.01, 2.44 ± 0.22, and 1.42 ± 0.028 mm/day with 6, 12.5, 25, and 50 μg/ml CH in the absence of strain BS001, while these were significantly (*P* < 0.05) raised, to respectively, 7.04 ± 0.05, 6.5 ± 0.05, 3.42 ± 0.02, and 1.78 ± 0.02 mm/day in the presence of *Burkholderia terrae* BS001 (**Figure 4**). Furthermore, the highest CH levels (i.e., 75 and 100 μg/ml) were initially detrimental to *Lyophyllum sp.* strain Karsten growth, even in the presence of *Burkholderia terrae* BS001. However, in the presence of *Burkholderia terrae* BS001, the fungus started to grow visibly after 21 day on the amended OFA plates and the presence of *Burkholderia terrae* cells had a large and significant positive effect on fungal growth rate afterward, being 0.74 ± 0.08 (75 μg/ml) and 0.25 ± 0.07 (100 μg/ml) mm/day. In the absence of the bacterium, fungal growth was completely abolished even 84–90 day following fungal inoculation (**Figure 4**).

We then evaluated the protective effect of *Burkholderia terrae* BS001 on *T. asperellum* 302 under CH pressure in OFA. First, similar to *Lyophyllum sp*. strain Karsten, CH at varying levels significantly inhibited the growth of *T. asperellum* 302 as compared to the zero-control (*P* < 0.05; not shown). No effect of the presence of *Burkholderia terrae* BS001 cells could be found at 50 and 75 μg/ml CH (*P* >0.05). However, at 100 μg/ml CH, strain BS001 cells again significantly protected the fungal hyphae (*P* < 0.05), resulting in a raised fungal growth rate (2.35 ± 0.09 mm/day) compared to the fungus growing without added bacterial cells (1.90 ± 0.07 mm/day).

***Effect on other fungi.*** The growth rates of* Rhizoctonia solani* AG3 and *F. oxysporum* pv *lini* FoLn3 on OFA plates under CH pressure were also evaluated in relation to the effect of *Burkholderia terrae* strain BS001. The growth of both fungi was significantly (*P* < 0.05) inhibited by the presence of 75 and 100 μg/ml of CH, whereas lower concentrations permitted fungal outgrowth rates similar to the (zero-CH) control. The growth of *Rhizoctonia solani* AG3, over 7 days, occurred at rates of 3.85 ± 0.212 and 4.75 ± 0.353 mm/day, in the presence of 75 and 100 μg/ml CH, while the growth rate was 8.5 ± 0.015 mm/day in the zero-CH controls. *Burkholderia terrae* BS001 did protect *Rhizoctonia solani* AG3 from the growth-inhibiting effect of CH to a significant extent (*P* < 0.05). In the presence of strain BS001, the fungal growth rates over 7 day were 5.15 ± 0.24 and 5.1 ± 0.08 mm/day, respectively, on OFA amended with 75 and 100 μg/ml CH, versus the aforementioned rates of 3.85 ± 0.21 and 4.75 ± 0.35 mm/day (Figure S4).

With respect to *F. oxysporum* pv *lini*, the growth rate over 7 days in zero-CH condition was 8.35 ± 0.01 mm/day, whereas it was significantly (*P* < 0.05) lowered, to 2.35 ± 0.212 and 2.03 ± 0.014 mm/day, under 75 and 100 μg/ml CH, respectively. In the presence of strain BS001, these growth rates were 2.75 ± 0.20 and 2.1 ± 0.08 mm/day at 75 and 100 μg/ml CH, respectively. Clearly, although these positive effects of strain BS001 persisted up to day 21, they were never significant (*P* > 0.05).

## DISCUSSION

Several members of the genus *Burkholderia,* including *Burkholderia terrae*, have been reported to be enriched in the mycosphere of fungi such as *Laccaria proxima* and *Lyophyllum sp*. strain Karsten in soil (Warmink and van Elsas, 2008, 2009). These *Burkholderia* strains apparently utilize the hyphal network that such soil fungi form as a colonizable surface (Warmink and van Elsas, 2009). In eco-evolutionary terms, we postulated in earlier work that the *Burkholderia terrae* and related bacterial types that were enriched from soil by the hyphae of soil fungi (in the mycosphere) may find their ecological niche at the surfaces of such fungi, as driven by nutritional and colonization demands.

A clear facet of the association of such bacteria with mycelial networks in soil is the fungal facilitation of passage of air-filled voids in the soil (De Boer et al., 2005). This allows soil bacteria, for which great barriers exist in terms of their movement through soil, to access and explore new microhabitats which may be millimeters to centimeters away (Kohlmeier et al., 2005; Furuno et al., 2010; Nazir et al., 2010). Interestingly, our prime bacterial migrator, *Burkholderia terrae* strain BS001, was shown to not only migrate itself but also to enable other bacteria, like *D. japonica* BS003, to move along the fungal highway (Warmink et al., 2011). This led to the hypothesis that local conditions, in the form of colonizable surface structures and/or water maintenance conditions, were modulated to such an extent that the movement of other bacteria that are able to make use of the modified conditions became possible. Such conditions are typically found at cell aggregates that may form at the fungal surface, a feature we showed earlier for the *Burkholderia terrae* BS001 – *Lyophyllum sp.* strain Karsten association (Warmink and van Elsas, 2009).

We here extend the detection of bacterial agglomerates along extending fungal hyphae in soil to a range of other fungi (**Figure 1**). By its capacity to live in close association with fungal hyphae and produce biofilm-type associations (as confirmed here for several fungal hosts), *Burkholderia terrae* BS001 has probably developed an optimal capacity to capture and utilize the compounds that are locally available, be these fungal-released or not. Using liquid media, strain BS001 has previously been found to induce the release of glycerol from the hyphae of *Lyophyllum* sp. strain Karsten, a compound that it can utilize for its own growth and survival (Nazir et al., 2013). However, to date we ignore the putative nutritional interaction of strain BS001 with the other test fungi used in this study.

Here, we provide evidence for the contention that *Burkholderia terrae* BS001 has a broad capacity to migrate along with the hyphae of a range of soil fungi, belonging to different genera and ecological classes, that are able to explore the (G) soil in the microcosm set-up that was used. Hence, the fungal-interactive functions of strain BS001, which may include cellular appendices such as the type 3 secretion pilus, flagella and type 4 pili (Haq et al., 2014), are not highly specific for just one colonizable fungal host. Moreover, the different fungi that were conducive to strain BS001 comigration allowed the outgrowth of the bacterium along with the fungal hyphae, albeit to different population densities. Clearly, next to the reference fungus *Lyophyllum* sp. strain Karsten, another four (out of six) fungal hosts allowed outgrowth of strain BS001 to high population densities. On the basis of the thus presumed broad fungal-interactive capacity, we surmised that *Burkholderia terrae* strain BS001 has evolved toward a superior fitness in terms of the exploration of soil microhabitats in conjunction with diverse soil fungi that are locally encountered. This broad capacity is likely to be multi-faceted and may include a diversity of bacterial mechanisms, as detailed in Haq et al. (2014). In a generic sense, it likely includes the physical ability of strain BS001 to produce biofilm-like cell agglomerates associated with the hyphal networks just like the recently reported association of *Bacillus subtilis* with *Aspergillus niger* (Benoit et al., 2014). Unfortunately, we could not show this to occur directly in the soil as a result of technical difficulties. However, using the simplified cover slip based “soil-derived” systems, cell agglomerates or layers were indeed found to be formed by strain BS001 at different fungi. Although we did not reveal fully blown biofilms, we hypothesize that the typical biofilm formation *pel* and *pga* gene clusters that were recently found to be present on the *Burkholderia terrae* BS001 genome (Haq et al., 2014) may have been involved.

Moreover, it was very interesting to observe that, whereas *Burkholderia terrae* BS001 on the one hand obtains a benefit from its association with a fungal host, on the other hand, it appears to protect its partner against antagonists like *Pseudomonas fluorescens* strain CHA0 and/or its metabolome, present in its microhabitat. In natural soils, many (bacterial or fungal) antagonists exist that can inhibit fungal growth and survival as a result of the production of secondary metabolites (Keel et al., 1992). Hence, our finding of protection against such agents by bacterial colonizers of fungal surfaces is of major importance, as it adds a concrete ecological phenomenon to existing theories about soil microbial interactomes.

To nail down the mechanism of protection, we performed experiments with the antifungal compound cycloheximide (CH), which can be produced in soil by bacteria of the genus *Streptomyces* (Abou-Zeid and El-Sherbini, 1975; Kominek, 1975a,b; Dykstra and Wang, 1990). The analyses indicated that *Burkholderia terrae* BS001 indeed can provide protection, potentially via shielding/sorption or detoxification effects, from CH to several of the fungi, at different levels of CH. Protection of fungi like *Rhizoctonia solani* AG3,* T. asperellum* 302, and/or *Lyophyllum sp.* strain Karsten by *Burkholderia terrae* BS001 against antifungal agents has great practical relevance. It may explain why, for example, plant disease management may fail or does not fully work in nature given the possibility that target plant-pathogenic fungi may have acquired bacterial protection much like that found in our study. Furthermore, and adding to the practical relevance, the efficiency of some biocontrol agents against plant disease might be improved by making use of a protective strategy, i.e., by combining a biocontrol fungus, such as the here-used *T. asperellum* 302, with the protective bacterium *Burkholderia terrae* BS001.

We propose that the protection is related to the activity of the cell agglomerates that are formed by *Burkholderia terrae* BS001 around the fungal hyphae. The bacterium, in its interactive behavior, when allowed to complete its associative process, may build a matrix composed of compounds such as extracellular polysaccharides at the fungal surface. Such a matrix may shield away some of the antifungal agents/compounds that are present in the vicinity. Alternatively, the antifungal agents are inactivated by a (bio)chemical transformation activity. Thus, the *Burkholderia terrae* strain BS001 cells around fungal mycelia may act as agents that counteract antifungal agents. The barrier could be physicochemical or biochemical, as antifungal compounds may be bound to, or inactivated by, the cell agglomerates at the fungus, thus becoming unavailable to the growing fungus. A potential effect of *Burkholderia terrae* BS001 on CH might be related to the local pH (data not shown), as CH may become unstable at higher pH levels (Lawes, 1962).

In the light of the current data on strain BS001 effects, we propose that (1) *Burkholderia terrae* BS001 is a generalist rather than a specialist in terms of its capacity to interact with mycelial fungi in soil, and (2) that its interaction with these soil fungi may be mutualistic rather than commensalistic. However, there is a paucity of knowledge on the mechanisms used by the bacterial as well as fungal partners in the associations. Hence, further in-depth research in this area is strongly advocated.

## AUTHOR CONTRIBUTIONS

Rashid Nazir and Jan Dirk van Elsas contributed to the conception and design of the work, drafting the work and revising it critically for important intellectual contents. Rashid Nazir and Diana I. Tazetdinova contributed in acquisition, analysis and interpretation of data for the work presented in this manuscript. Jan Dirk van Elsas and Rashid Nazir collectively worked on final approval of the version to be published. All three authors agreed to be accountable for all aspects of the work for the accuracy and/or integrity.

## Conflict of Interest Statement

The authors declare that the research was conducted in the absence of any commercial or financial relationships that could be construed as a potential conflict of interest.

## Abbreviations

CH: cycloheximide
OFA: oat flake agar
cm: CHA0 metabolites
cmT: CHA0 metabolites with *Trichoderma*
