# Supplementary material for: Burkholderia terrae BS001 migrates proficiently with diverse fungal hosts through soil and provides protection from antifungal agents
Source: Front Microbiol. 2014 Nov 11;5:598. doi: 10.3389/fmicb.2014.00598 (PMC4227525; doi:10.3389/fmicb.2014.00598)
Supplement: Supplementary file 1 [file Presentation_1.PDF]

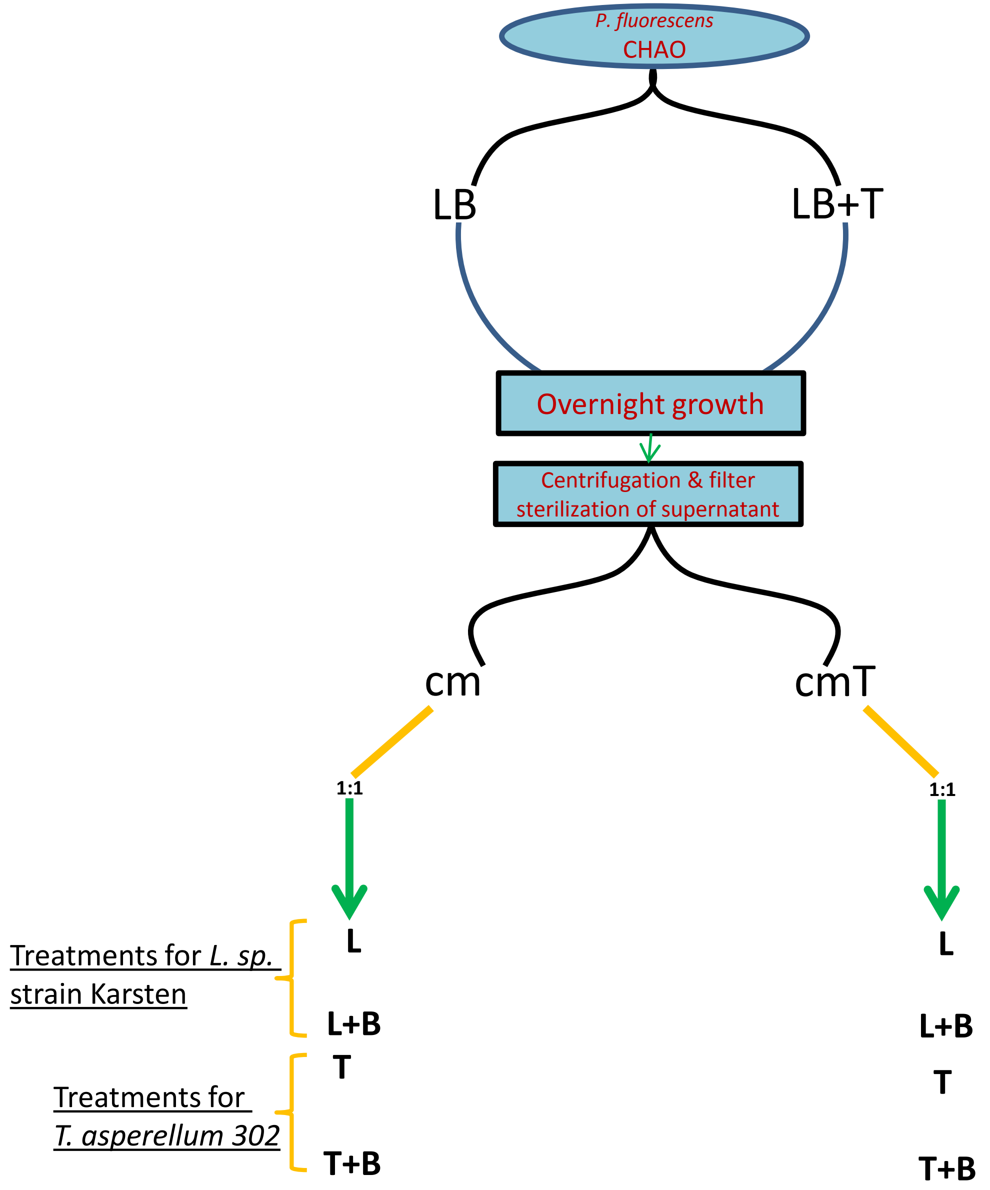

Fig. S1a. Schematic representation of the procedure to produce metabolites of *P. fluorescens* CHA0 and their use against fungal growth. LB, CHA0 growing singly in LB broth; LB+T, CHA0 and *Trichoderma asperellum 302* together. cm, CHA0 metabolites (supernatant) when growing alone; cmT, CHA0 metabolites in the presence of *T. asperellum*. 1:1 means the ratio of concentration of CHA0-supernantant (metabolites) in OF-medium.

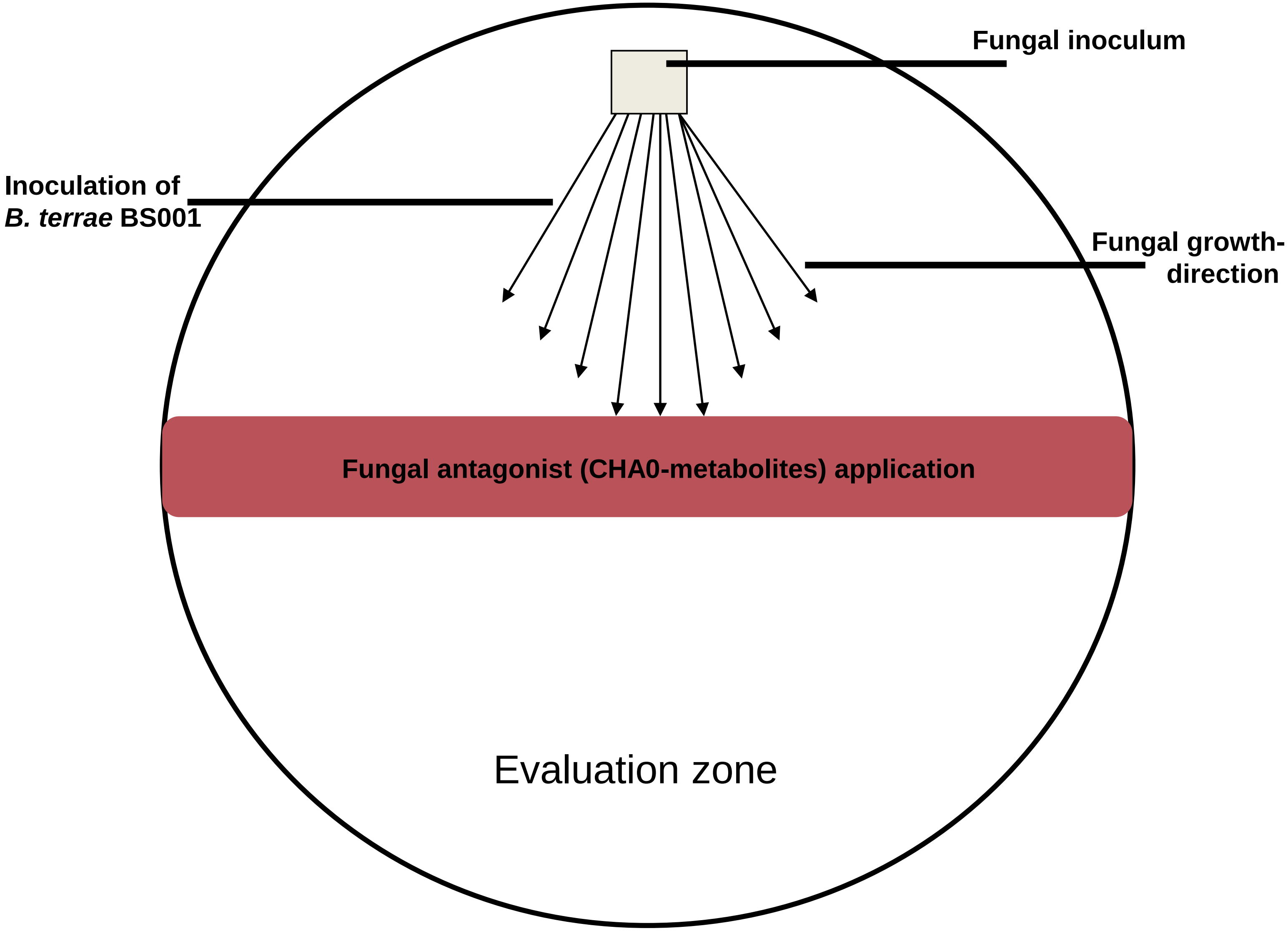

Fig. S1b Schematic representation of the procedure of application of metabolites of *P. fluorescens* CHA0 against fungal growth. The reddish stripe was cut out from the fungal growth (oat flake) agar and water agar + CHA0 supernatant (1:1) was re-poured in the stripe.

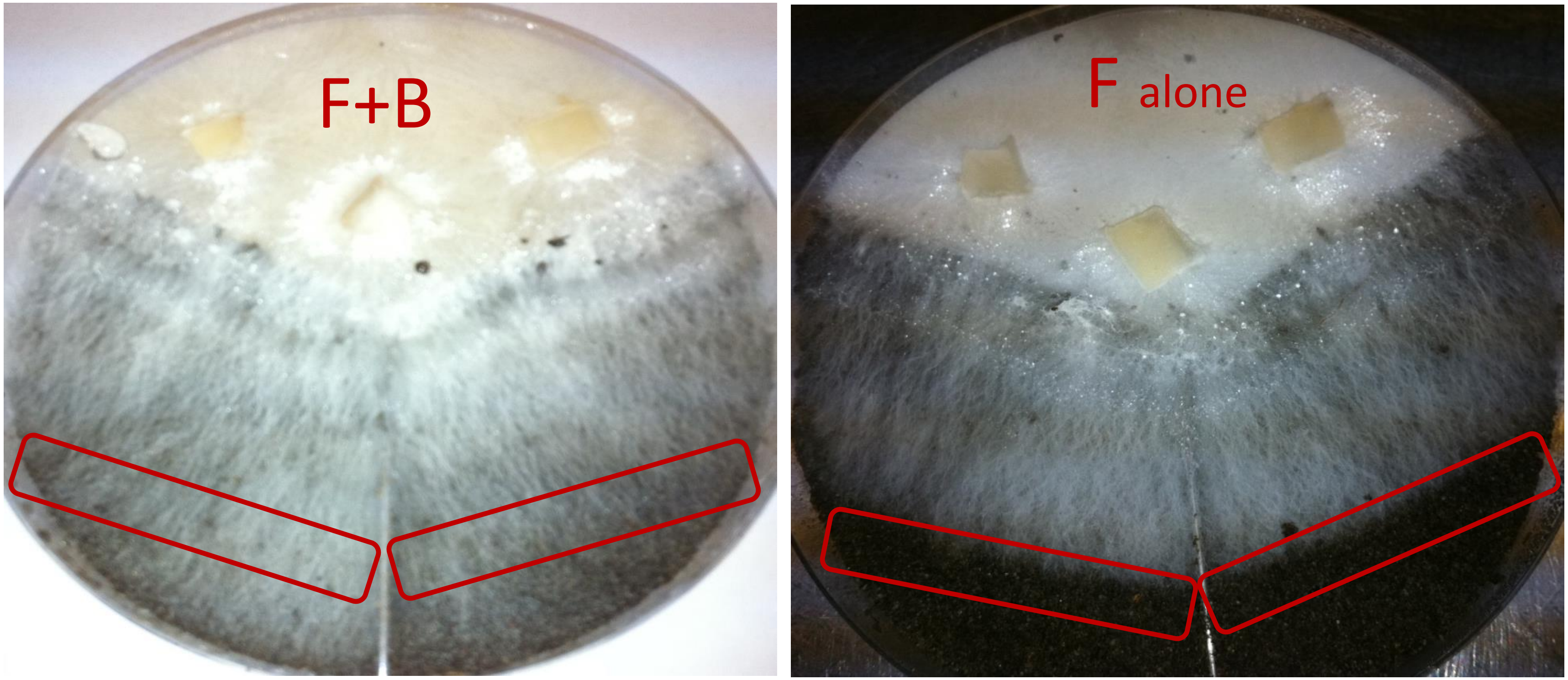

Fig. S2a Protection effect provided after 15 days by *Burkholderia terrae* BS001 to *L. sp*. strain Karsten against *P. fluorescens* CHA0**.** Left panel: strain BS001 present; right panel: BS001 absent. Red blocks indicate the place in soil microcosm where the antagonistic agents , i.e. CHA0 were applied.

25 µg/ml CH in G soil

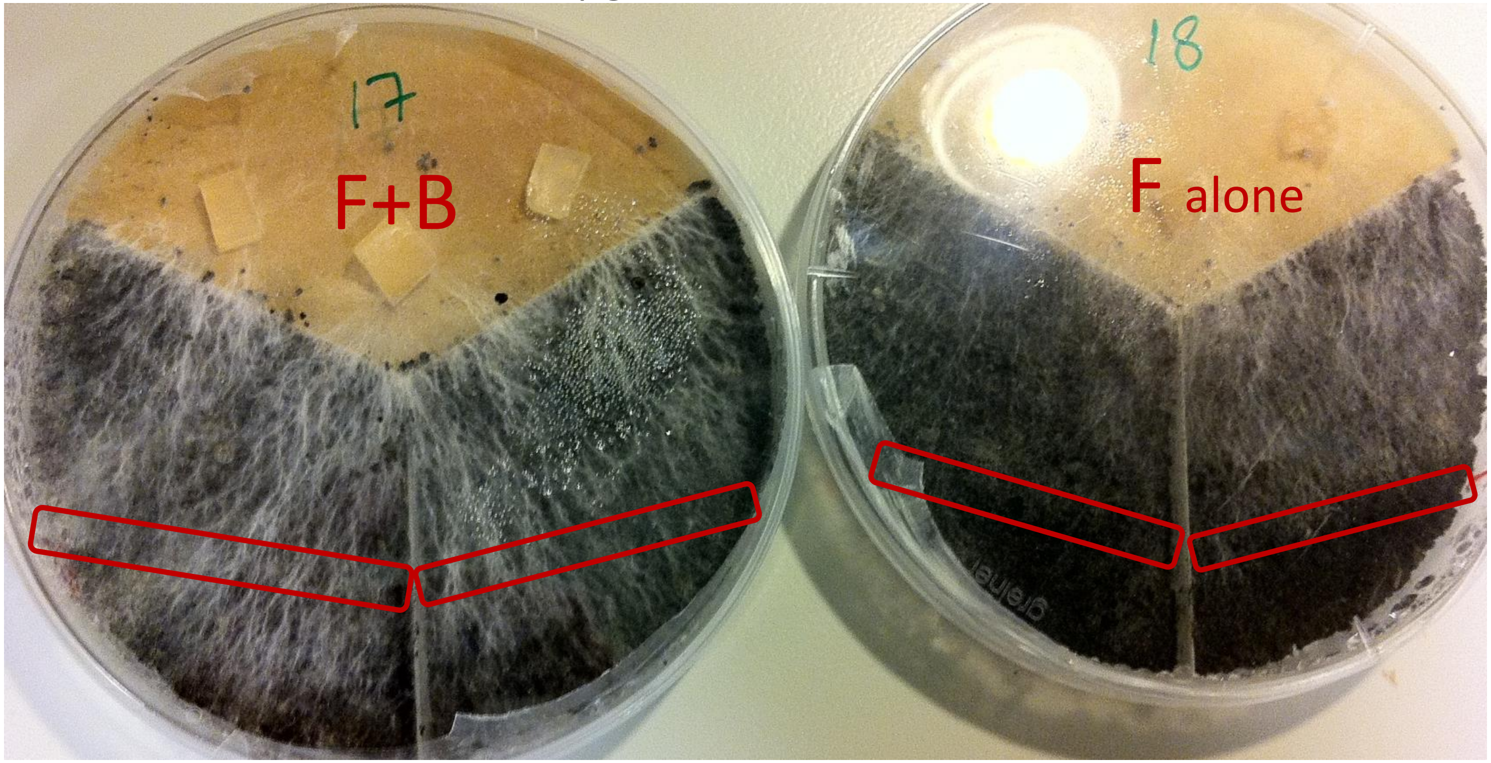

Fig.S2b Protection effect provided after 15 days by *Burkholderia terrae* BS001 to *L. sp*. strain Karsten against CH (25µg/ml). Left panel: strain BS001 present; right panel: BS001 absent. Red blocks indicate the place in soil microcosm where the antagonistic agent, i.e. CH was applied.

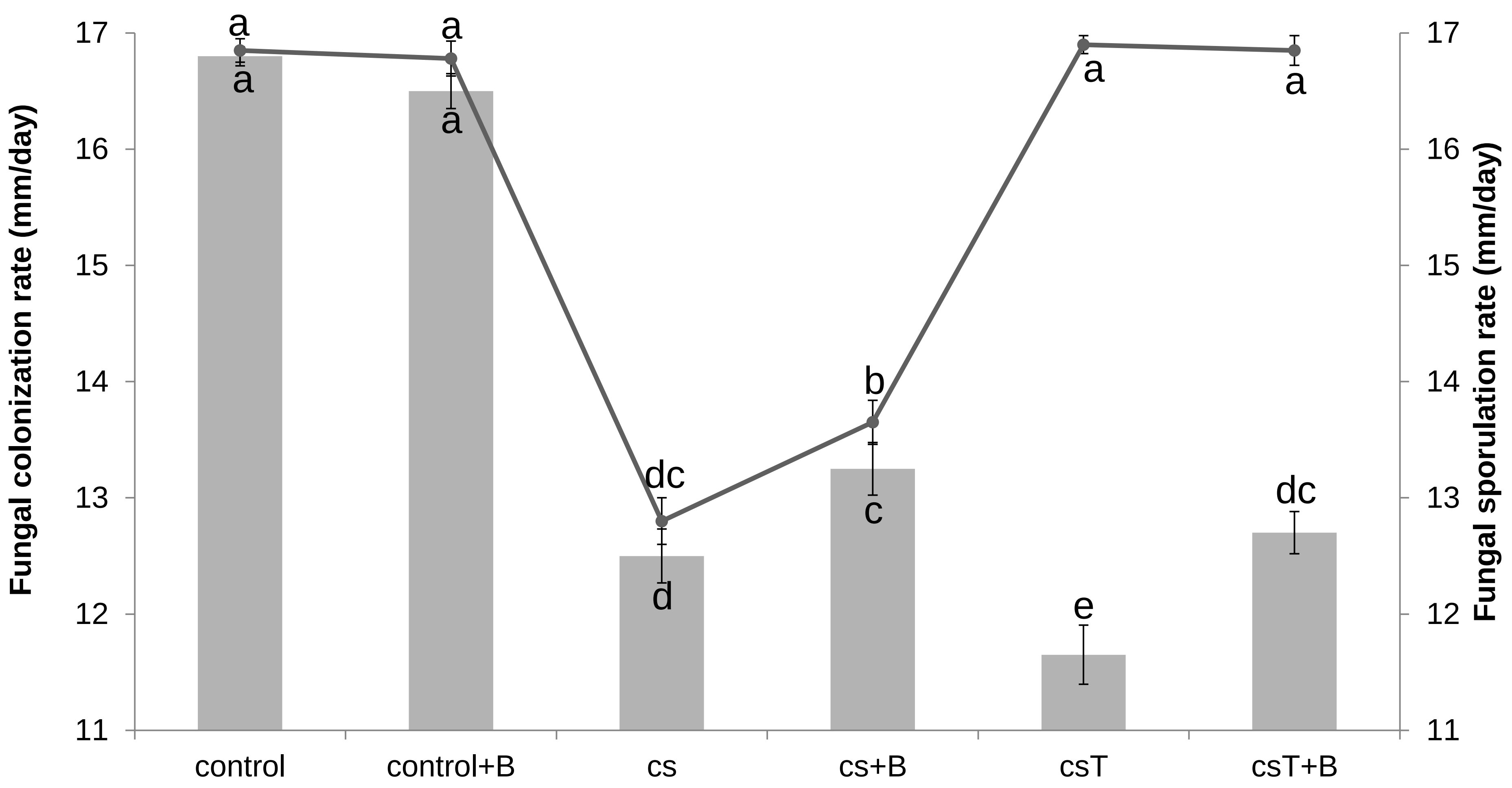

Fig. S3. *Trichoderma asperellum 302* growth and sporulation response observed after 5-days confrontation with *P. fluorescens* CHA0 metabolites in OFA. cm, OFA+ CHA0 metabolites in LB; cmT, OFA+ CHA0 metabolites when grown in LB along with *Trichoderma asperellum 302*; cm+B and cmT+B, aforementioned OFA media, using *B. terrae* BS001 co-inoculated on the same day. Light bars: sporulation (left Y-axis); lines: hyphal (colony) growth (right Y-axis) of *T. asperellum* 302.

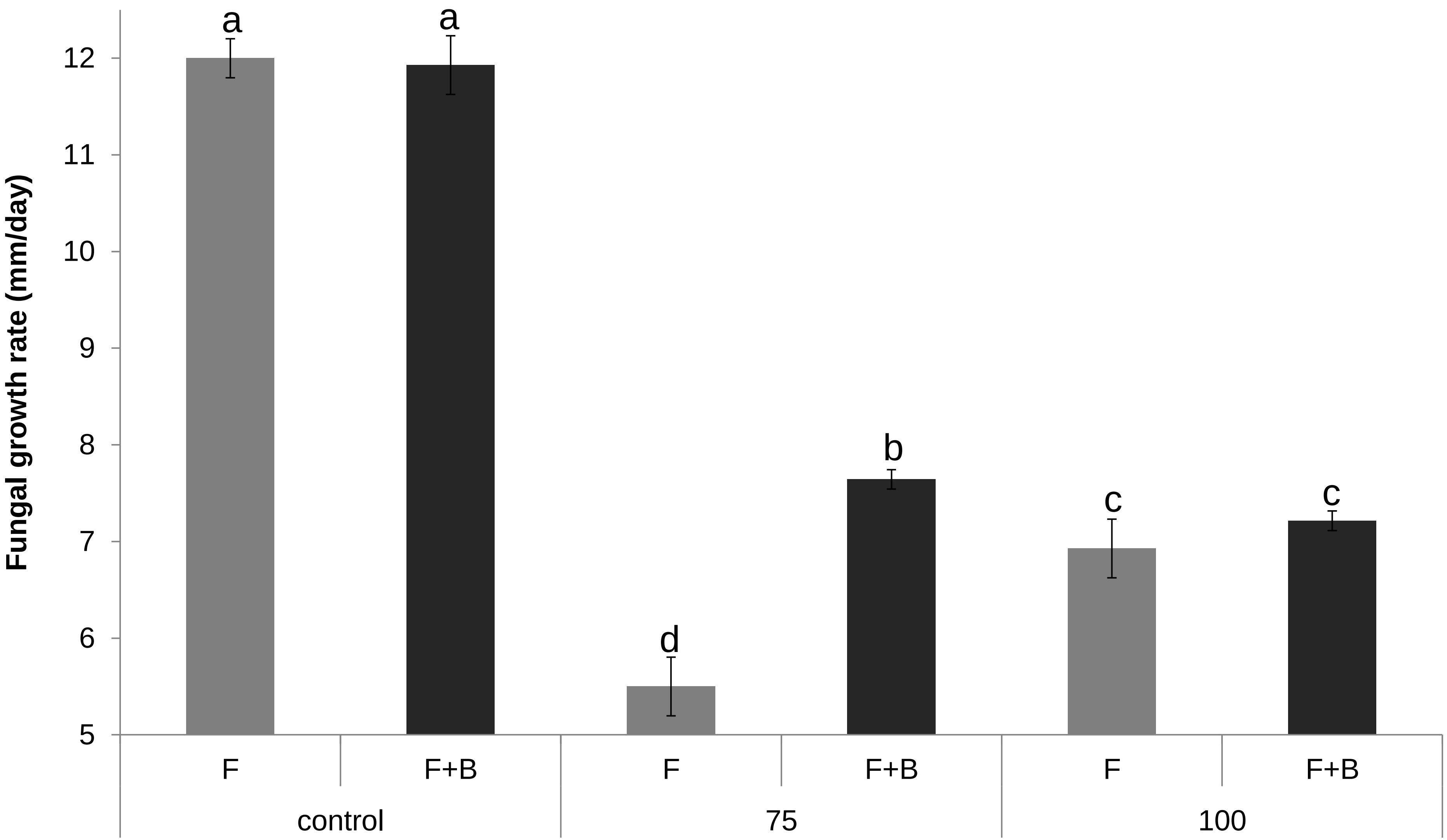

Fig. S4 Protective effect of *B. terrae* BS001 observed after 7 days to *Rhizoctonia solani* AG3 against CH. F, *R. solani* AG3; B, *B. terrae* BS001. 75 and 100: CH concentrations (µg/ml) in OFA; control: no addition of CH in OFA.
